# Using familial information for variant filtering in high-throughput sequencing studies

**DOI:** 10.1007/s00439-014-1479-4

**Published:** 2014-08-17

**Authors:** Melanie Bahlo, Rick Tankard, Vesna Lukic, Karen L. Oliver, Katherine R. Smith

**Affiliations:** 1 The Walter and Eliza Hall Institute of Medical Research, Parkville, VIC 3052 Australia; 2 Department of Medical Biology, University of Melbourne, Melbourne, VIC 3010 Australia; 3 Department of Mathematics and Statistics, University of Melbourne, Melbourne, VIC 3010 Australia; 4 Epilepsy Research Centre, The University of Melbourne, Austin Health, Heidelberg, VIC 3084 Australia

## Abstract

High-throughput sequencing studies (HTS) have been highly successful in identifying the genetic causes of human disease, particularly those following Mendelian inheritance. Many HTS studies to date have been performed without utilizing available family relationships between samples. Here, we discuss the many merits and occasional pitfalls of using identity by descent information in conjunction with HTS studies. These methods are not only applicable to family studies but are also useful in cohorts of apparently unrelated, ‘sporadic’ cases and small families underpowered for linkage and allow inference of relationships between individuals. Incorporating familial/pedigree information not only provides powerful filtering options for the extensive variant lists that are usually produced by HTS but also allows valuable quality control checks, insights into the genetic model and the genotypic status of individuals of interest. In particular, these methods are valuable for challenging discovery scenarios in HTS analysis, such as in the study of populations poorly represented in variant databases typically used for filtering, and in the case of poor-quality HTS data.

## Introduction

High-throughput sequencing (HTS) has proven to be highly successful at identifying causal variants for many genetic disorders with a variety of underlying genetic etiologies (Boycott et al. 2013). This includes genetic disorders segregating in clear dominant and recessive inheritance modes, but also disorders that are caused by less well-studied genetic models such as de novo mutations. HTS has also been successfully applied to small cohort studies, even in the presence of locus heterogeneity (Riviere et al. 2012). HTS can potentially identify all causal variants, hence cohorts of unrelated individuals with different causal alleles are very powerful for the implication of a causal gene since the accumulation of rare, likely pathogenic variants in a single gene, gene family or pathway, is highly unlikely, even for just a few affected individuals.

**Fig. 1:**
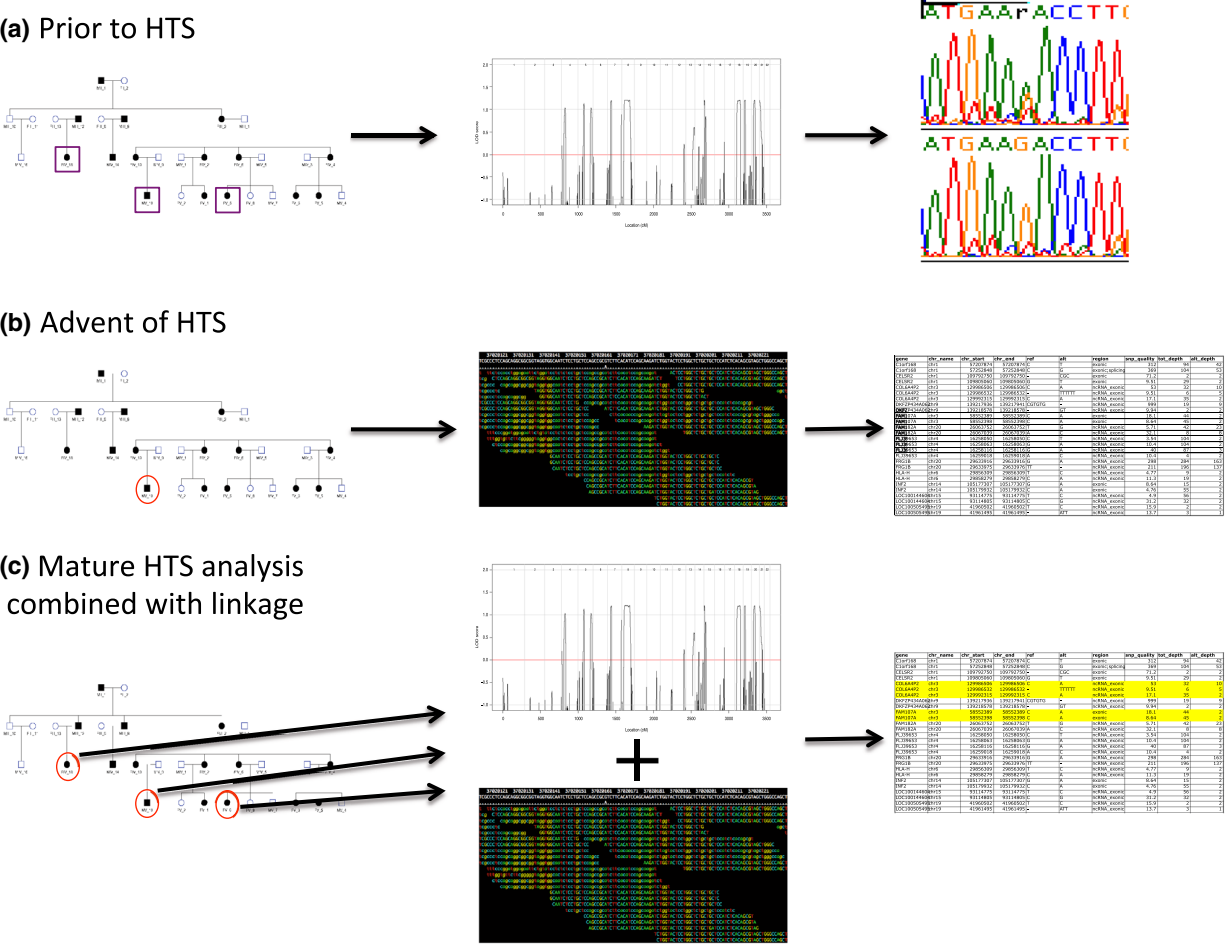
Identification of a causal variant in a family study, **a** prior to the advent of HTS, using linkage and Sanger sequencing, **b** advent of HTS, using only a proband, or one or two individuals from a family, ignoring relationships between the individuals, leading to lengthy lists of putative causal variants, and **c** mature HTS analysis, where family information is incorporated using genotypes derived from HTS data to perform a linkage or IBD analysis, leading to a much reduced list of variants

HTS has identified variants that cause germline Mendelian disorders (Ng et al. 2010a, b) segregating in a highly penetrant manner, expediting the discovery of many such variants (Heron et al. 2012). Such diseases often come to notice because they segregate in one or more families. Prior to the HTS era, linkage analysis, or other identity by descent (IBD) approaches were applied to identify genomic regions of interest where the affected status co-segregates with genotypic status. The application of HTS has resolved several such Mendelian disorders that had achieved promising linkage results, including highly significant linkage results (parametric LOD scores >3), and were awaiting causal gene discovery (Corbett et al. 2010, 2011; Koenekoop et al. 2012; Reversade et al. 2009). As such, identification of a causal gene for a genetic disorder before the advent of HTS usually required a two-step process, with the first step being a genome-wide, or chromosome-wide, localization step, utilizing IBD information inferred through co-segregation of genetic markers such as microsatellites and single-nucleotide polymorphisms (SNPs) with disease (linkage analysis). The second step consisted of the actual identification of the causal variant with Sanger sequencing making this task manageable by focusing the search on a particular genomic region, usually only a few Megabases in length. Genome-wide significant linkage was usually required before traditional Sanger sequencing commenced, but without any obvious candidate genes this could then lead to the laborious sequencing of all genes (50–100, depending on the size and location of the linkage region) in the region, potentially taking years before a causal variant and gene were found. HTS has led to an expedition of the discovery of causal variants by circumventing the gene by gene sequencing step (Fig. 1). Furthermore, since the HTS approach is now most cost-effectively performed genome-wide, via approaches such as whole exome sequencing (WES), the first step of genomic localization has been seen as unnecessary and more often bypassed. Probands of small families may then be contributed to consortium studies where they are treated as singletons, ignoring the family information.

HTS has also been applied successfully to identify causal mutations in a number of different genetic models including those not previously amenable to analysis. This includes de novo germline mutations (Epi4K Consortium et al. 2013; Jiang et al. 2013; Lim et al. 2013). Sporadic mutations benefit from the application of the trio approach, where variants inherited from the parents are eliminated to identify the novel mutations in the proband. This has led to new insights into the genetic basis of complex disorders such as autism and other neuropsychiatric disorders, leading to further debates about the role of such variants in heritability calculations (Gratten et al. 2013). Sporadic somatic variants (Baek et al. 2013; Lee et al. 2012; Poduri et al. 2012) have also been identified. This is another genetic model that was intractable prior to HTS and highlights the possibility that somatic variants may play a hitherto underestimated role in causing genetic disease outside the cancer arena.

In germline Mendelian disorders HTS has been most successful for recessive disorders, in particular autozygous disorders, which are caused by single mutations. Causes of dominant disorders continue to be more resistant to discovery for several reasons. Heterozygous variants are harder to call as variants than homozygous alternative variants because the binomial variation of reads with particular alleles is higher for the heterozygote than for the two homozygous states. Furthermore, individuals have more heterozygous than homozygous novel rare variant alleles, making it more difficult to filter out novel benign polymorphisms. This is particularly problematic in populations that are not well represented in databases, where even large numbers of novel, nonsynonymous autozygous variants may remain (Azmanov et al. 2013) after filtering using standard databases. Proof that such discovered variants are not population polymorphisms can require the sequencing of many candidate variants in a relevant population sample which is time consuming and costly.

The advent of HTS saw the need for the development of pipelines to handle the tens of thousands of variants that are routinely called in human genomes. These pipelines are still mainly based on single sample/individual analysis, hence the incorporation of familial information is not routine. Even when familial information has been available for use in conjunction with HTS data it is not always used, despite the potential for IBD or linkage information to add a powerful filter for detected variants. Familial information is often included in a very indirect way by applying a genotyping filter, based on an inferred genetic model from the pedigree, with specific genotype assumptions for unaffected individuals, carriers, non-carriers and affected individuals.

HTS studies are highly reliant on ascribing likely pathogenicity according to mutational databases such as the Leiden Open Variation Database (LOVD, http://www.LOVD.nl/), The Human Gene Mutation Database (HGMD, http://www.hgmd.cf.ac.uk/) and ClinVar (https://www.ncbi.nlm.nih.gov/clinvar/). However, these databases are known to be incomplete, missing known mutations. Additionally, Bell et al. (2011) showed that 25 % of variants, described as causal in mutation databases, were low frequency polymorphisms and not causal, highlighting the need to assess candidate variants further, gathering additional evidence for likely causality. Early HTS studies were not always required to provide additional data to support variant discoveries, such as multiple mutations in the same gene or functional work or evidence of causality in an experimental model (Piton et al. 2013). Hence, there is a need to reconsider current HTS analysis methodology. One way forward is to make greater use of additional information such as that afforded by familial information to provide greater protection from false positive findings. Many more HTS studies remain unpublished because a causal variant has failed to be identified. These studies may benefit from the approaches discussed here, potentially highlighting a hitherto unpromising variant due to the current, perhaps limited, understanding of the gene’s biological role, or at least highlighting regions in the genome that require greater scrutiny, perhaps with supplemental sequencing, or generation and analysis of a second attempt at WES, with a newer, more comprehensive exome capture platform, or performing WGS.

Here, we describe a set of observations for the analysis of both single and familial HTS data that can and should be used to help in the identification of the causal variant. Making use of familial and population-based information for HTS data helps by: (1) extending quality control (QC) steps, (2) identifying IBD regions, (3) validating the genetic model, and (4) validating haplotype segregation by making use of IBD information, with or without additional SNP chip data. Using this type of information can lead to the accumulation of evidence in favor of particular variants, determine the correct genetic model, lead to the elimination of irrelevant variants and can also narrow the search space for variants to particular genomic regions.

HTS data is highly non-uniform in coverage which is influenced by GC content of the underlying sequence where both very high and very low GC content DNA sequence is captured by fewer HTS reads (Dohm et al. 2008). In addition, this known effect can be exacerbated by technology such as exome capture, or by additional biological signals such as DNA source. An example of the latter is the DNA fragmentation present in cell-free DNA which displays DNA motif bias (Chandrananda et al. 2014). This bias leads to areas of the genome that are difficult to interrogate with HTS which may require higher coverage, multiple HTS applications or necessitates improvements in HTS technology to be able to be sequenced to a sufficient depth to detect variants. Thus, by identifying regions of the genome that have been detected as IBD researchers become aware of regions that deserve greater scrutiny, possibly also for more unusual forms of genetic variation.

Therefore, rather than being relegated to history, IBD methods should be recognized as providing a powerful ally for HTS to help identify causal variants. We wish to remind researchers of the availability of these approaches, outlining some resources, which could be applied to help solve their HTS study.

## Extracting HapMap SNPs from HTS data

Many HTS studies are applied to multiple related individuals, with filtering of variants from all the individuals, ignoring familial relationships. One reason for this approach and its increasing popularity is the decreasing cost and bulk production of HTS data. This often sees researchers forgoing SNP chip data generation and traditional linkage mapping approaches, even when the family has sufficient power to produce genome-wide significant linkage. However, it is possible to extract SNPs from HTS data by instructing variant callers to genotype variants at HapMap SNP positions to create pseudo SNP chip data for all individuals that underwent HTS. Hence linkage analysis and other analyses can still be performed successfully.

**Fig. 2:**
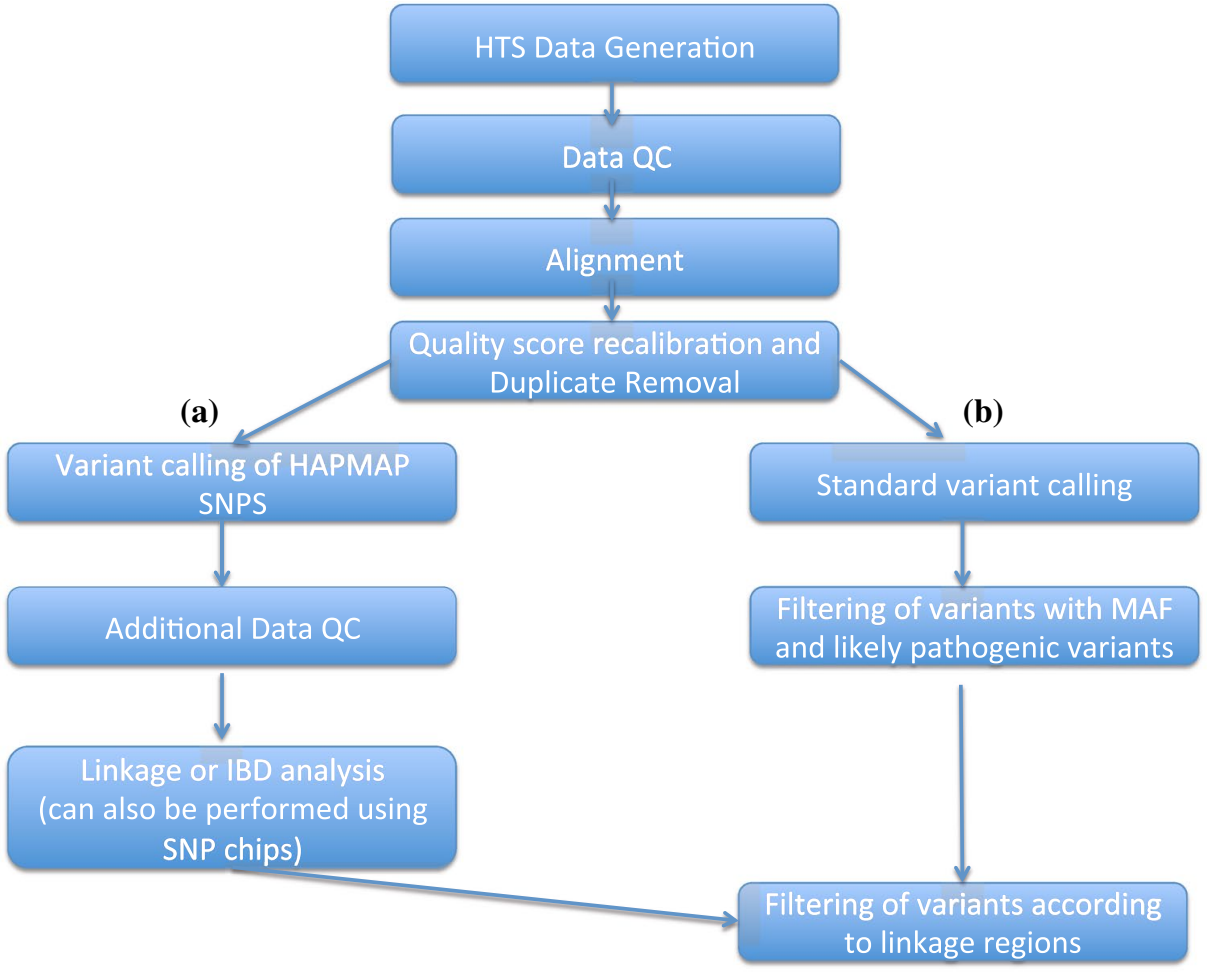
Dual workflow for HTS data analysis that takes advantage of HapMap SNPs for linkage mapping (**a**), and then uses this information in the filtering steps in (**b**)

Instructions on how to call HapMap SNPs from HTS data, including methods to process this data into linkage and IBD analysis ready files can be obtained via http://bioinf.wehi.edu.au/software/linkdatagen. The methods are described in Smith et al. (2011) and Bahlo and Bromhead (2009). Any genome-wide (or chromosome wide) HTS data suffices, as long as enough HapMap SNPs are covered sufficiently to allow genotype calling. The workflow required is more complicated but is not difficult to implement (Fig. 2).

WES by definition captures mainly exonic DNA. Exonic regions are known to have more SNPs that undergo balancing selection leading to lower MAFs on average (1000 Genomes Project Consortium et al. 2010), thus genotyping data from WES will not be as useful for IBD and linkage analysis as SNP chips or even genotyping data extracted from comparable median coverage WGS to the median coverage WES data (targeted areas only). Table 1 outlines the numbers of HapMap SNPs that are called with ≥10× coverage in more than 50 % of samples using one WES capture platform in current use (Agilent V5 + UTR). This shows the availability of tens of thousands of SNPs for linkage and IBD analysis. Many IBD and linkage analyses make use of only ~10,000 SNPs due to the constraints of linkage equilibrium that are imposed to satisfy the first-order Markov models that underpin most of these methods. In Smith et al. (2011), we compare genome-wide LOD scores derived from SNPs genotyped from the Illumina TruSeq capture platform to those derived from a high-density SNP chip, showing very few differences in the linkage analysis results. Inbreeding inference utilizing HTS genotypes is also possible with FSuite (Gazal et al. 2014), showing good performance.

**Table 1:** HapMap SNPs available for linkage and IBD analysis based on ≥10× average coverage of the targeted regions in at least 50 % of samples

Population	Available SNPs	Covered SNPs	% of total SNPs	Number SNPs with Het >0.3	% of covered SNPs
CEU_2	3801563	153388	4.03	50231	32.75
CHB_2	3827537	153484	4.01	47498	30.95
JPT_2	3827726	153689	4.02	47351	30.81
YRI_2	3750555	152239	4.06	51241	33.66
CEU_3	1520715	88970	5.85	39174	44.03
ASW_3	1463106	83303	5.69	41279	49.55
CHB_3	1519591	88653	5.83	36806	41.52
CHD_3	1246085	69281	5.56	35887	51.80
GIH_3	1337706	74005	5.53	39370	53.20
JPT_3	1518437	88455	5.83	36720	41.51
LWK_3	1440446	83045	5.77	39271	47.29
MEX_3	1380212	79198	5.74	38910	49.13
MKK_3	1451099	81916	5.65	40321	49.22
TSI_3	1347642	74645	5.54	38875	52.08
YRI_3	1520811	89382	5.88	39684	44.40

The selected SNPs are over the four HapMap Phase II populations (designated by a _2) and the eleven HapMap Phase III populations (designated by _3). Data is based on the analysis of 20 Agilent V5 + UTR WES captured samples that were sequenced with Illumina HiSeq 2000 sequencing at the Australian Genome Research Facility, Melbourne, Australia

## Additional quality control steps with familial HTS data

The bulk processing and high-throughput nature of HTS usually necessitates batching of samples, which is often accomplished through molecular barcoding. The generation of input for HTS platforms is thus a source of potential error with sample mix-ups or mishaps in the sequencing library preparation step that can lead to contaminated samples. As such it is important to check sample veracity prior to analysis. The ability to extract genotype information allows an expanded repertoire of error checks that make use of the genotyping calls. Mendelian error detection, gender estimation and population-based checks, based on the genotyping data, can identify sample mix-ups, pedigree misspecifications and sample contamination. We now describe these in further detail.

## Variant calling and Mendelian error checking

HTS analysis usually proceeds through standard pipelines such as those described in Altmann et al. (2012), which are predicated on single samples. We recommend calling samples to be analyzed as a genetic entity (single or multiple families) jointly, as this can increase power to identify rare variants. Furthermore, using a family-oriented HTS analysis pipeline can also produce Mendelian consistent genotype data from HTS data by making use of variant callers that incorporate familial information. FamSeq (Peng et al. 2013) and PolyMutt (Li et al. 2012) improve variant calling by making use of familial information while MATE-CLEVER (Marschall et al. 2013) performs family aware indel detection and calling. Once HapMap SNP genotypes are called, Mendelian error checking can be performed via LINKDATAGEN (nuclear family Mendelian error checks) or through even more sensitive Mendelian error detection programs such as PEDCHECK (O’Connell and Weeks 1998) and sophisticated pedigree misspecification analysis such as PREST (Sun et al. 2002) where Mendelian errors are utilized to check specified pedigree relationships. LINKDATAGEN also includes a population-based test for samples that assesses the fit of the three classes of genotype calls (homozygous reference, heterozygote, homozygous non-reference allele) against the expected values for all known HapMap populations. This test not only highlights whether an assumed population is correct but can also detect sample contamination that can occur with HTS data.

Further error checking is performed by specific linkage analysis and IBD sharing programs that can detect errors or incorporate errors into analysis. This is more important for HTS-generated genotypes since genotyping errors can be much higher than for SNP chip-derived genotyping data. The error rates depend on the thresholds placed on called variants that have to be set taking into account the median coverages achieved in the family study and the robustness of the following analyses to genotyping errors. Further error checks using probabilistic approaches make use of expected versus observed recombination events, such as those implemented in MERLIN (Abecasis et al. 2002), and are able to detect additional genotyping errors that can also be removed.

One important benefit of linkage analyses in families is that more missingness and error can be tolerated than in pairwise IBD analysis since much information can be often imputed, with little uncertainty. We performed an analysis on a pedigree of 15 WES samples with a high level of missingness (median of medians of coverage 3, range [2, 9]) due to poor WES data generation, yet we were still able to detect a linkage peak and rapidly identify the causal variant despite the inability to identify the variant in many samples due to low coverage (Eggers et al. 2014).

## IBD and linkage analyses

IBD analyses are usually performed pairwise, either with known or unknown relationships between the two individuals. IBD analysis is often performed agnostic to the disease status, although a typical application is to assess IBD sharing between affected individuals. In the case of a known relationship between a pair of individuals IBD calculations simply calculate the probability of the observed genotyping data given the relationship, where the number of meioses separating the two individuals determines the rate of change of the underlying (hidden) IBD process. IBD can be calculated as a genome-wide summary, or at specified locations in the genome. In contrast, linkage analysis is a likelihood ratio test statistic that compares the probability of the genotyping data, for possibly more than two individuals, under two different hypotheses. The null hypothesis calculates the probability of the data assuming no linkage between the phenotype (modeled as another genetic marker) and the genotyping data from many genetic markers, and compares it to the alternative hypothesis of linkage between the phenotype and the set of genetic markers. This function of the likelihood ratio is known as the LOD score and can be calculated at any position in the genome.

Linkage and IBD analyses are powerful approaches for the identification of genomic regions harboring the causal variants for Mendelian diseases. Thus, both linkage and IBD methods are useful tools for filtering detected variants in cohorts of related individuals with HTS data. To implement this, one performs IBD or linkage analysis as described above, with or without SNP chip data, prior to variant filtering. These regions are then used as an additional filter for variants that remain to be considered. Other authors have designed genetic model-specific hidden Markov models for IBD detection (Eggers et al. 2014; Krawitz et al. 2010; Roach et al. 2010; Rodelsperger et al. 2011). For autozygosity mapping some authors have made use of simple homozygous run searches, focusing their search in these regions (Bilguvar et al. 2010). Extracting HapMap SNPs and their genotypes from HTS data allows a much broader usage of SNP genotypes derived from these studies and enables the use of well-understood statistical models and existing algorithms and software for IBD and linkage analyses (Tsoi et al. 2014).

Linkage analysis not only identifies linkage regions but allows post hoc analysis of the best inferred haplotypes, generated by programs such as MERLIN. This is achieved with graphical software such as HaploPainter (Thiele and Nurnberg 2005), by examining regions of interest. Individuals that violate the assumed genetic model can be identified, such as phenocopies or incompletely penetrant individuals. Once a susceptibility haplotype is identified, it is then possible to filter the variants based on matching of the genotypes called for the variant to the susceptibility haplotype status, rather than the underlying genotypes proposed by the original phenotype. An example of where such an approach is valuable is a dominantly inherited phenotype with age-dependent penetrance, but fully penetrant otherwise. Consider a younger, unaffected individual. This individual is identified as having the susceptibility haplotype, based on the linkage analysis. Thus, if this individual is sequenced it is appropriate to seek variants that are heterozygous for this individual. However, for most rare diseases the phenotype and susceptibility haplotype carrier status will match the hypothesized genetic model. If the WES data is poor, resulting in high levels of missingness, it is important to acknowledge that the inferred haplotypes conditional on the data will likely be non-unique, thus sampling from the posterior probability and examining a few sets of haplotypes is a useful supplementary approach. This can be achieved using the --sample option in MERLIN. We used this approach in Eggers et al. (2014) to verify the robustness, or lack thereof, of the inferred haplotypes. The uncertainty in the haplotypes does not have as much impact on the calculation of the LOD scores since these are summed over all possible haplotypic states that fit the data.

With the advent of dense SNP genotyping data, such as that observable in SNP chip or HTS data, it is possible to detect relatedness between individuals (Purcell et al. 2007), without prior genealogical information, including the presence of consanguinity (Albrechtsen et al. 2009; Leutenegger et al. 2006). This relatedness inference can then be included in subsequent linkage or IBD analysis (Guergueltcheva et al. 2012; Ivanov et al. 2014). For a review of methods that are applicable for this problem see Browning and Browning (2012).

We, and others, have successfully applied this approach to HTS data, detecting inbreeding and thus making powerful use of small families (Browning and Browning 2012; Guergueltcheva et al. 2012; Smith et al. 2012, 2013). The identification of autozygous regions, even after discovery of rare or novel homozygous variants, help to provide further evidence that the variants are in fact causal with variants that are merely homozygous by state and not homozygous by descent (autozygous) being able to be filtered out.

The methods outlined here use IBD analysis to lead to a discretized, independent filter of variants. Ideally, one would like to utilize the IBD information as weights rather than this discretized approach. Two recently published methods are the first to describe such approaches (Koboldt et al. 2014; Santoni et al. 2014). This will be useful in families where the genetic model is uncertain due to incomplete penetrance or possible phenocopies and vital for studies and pedigrees where strict Mendelian inheritance does not apply.

## Compound heterozygote filtering in recessive diseases

Once a region of interest has been identified and the haplotypic status of the sequenced affected and unaffected individuals ascertained, it may be possible to further interrogate variants under certain genetic models. One such model is the recessive compound heterozygote model where affected individuals have inherited one defective allele from each parent; one located on a maternally derived haplotype, and the other defective allele located on a paternally inherited haplotype. Thus, any two causal alleles need to be in ‘trans’, that is, on different haplotypes within the same gene. Genes that only contain rare or novel variants that are all in ‘cis’ may then be filtered out. This can be assessed in two ways.

The first method is contingent on the availability of parental genotypes and allows the phasing of each variant using the trio approach where one checks that each of the two variants being interrogated is from one parent precisely. This can be done using a simple SNP by SNP approach or can be performed using inference of inherited haplotypes to infer where recombinations between grandparental haplotypes have taken place. This requires a hidden Markov Model that allows for linkage disequilibrium and has been implemented using a population-based approach by Delaneau et al. (2013) for HTS data and in a family-based approach in MERLIN (Abecasis and Wigginton 2005), for genotyping data processed with LINKDATAGEN. These two methods can be used even in the absence of parental data. The Delaneau et al. (2013) method uses a property of HTS data: each read and read-pair fragment is a mini haplotype and these mini-haplotypes can be strung together (assembled), similar to de novo assembly, to inform whether variants are in the desired ‘trans’ or incorrect ‘cis’ state. This method is useful when parental information is absent, or only partially available, and thus uninformative for haplotype of origin based on the SNP by SNP phasing in trios method already described. To implement the method one can use the ReadBackedPhasing tool from the GATK toolkit (DePristo et al. 2011), HapCUT (Bansal and Bafna 2008) or HapCompass (Aguiar and Istrail 2012). The likelihood of homozygous parents is small for rare variants but is likely to also be more important when the individuals are from populations underrepresented in variant databases, where more of these types of variants will be observed.

## More unusual sources of genetic variation

Standard HTS pipelines call single-nucleotide variants (SNVs) and small indels only (those that can be detected with a simple HTS analysis pipeline). The identification of moderate-sized indels require additional analysis of the HTS data with specialized algorithms such as Pindel (Ye et al. 2009). Analysis of larger CNVs also require nonstandard pipeline analysis, since they depend either solely or at least partially on depth of coverage (Alkan et al. 2011). The detection of structural variation other than CNVs, for example translocations, is also possible (Chen et al. 2009). All of these variants can be heritable and causal for genetic disorders. The identification of IBD regions or linkage regions can indicate where standard pipelines have probably failed or need to be enhanced by looking for these alternative, less common, sources of disease causing variants. Many of these detection methods are still under development or prone to false positives and negatives, thus will benefit from fine tuning such as that afforded by being able to focus on specific genomic regions.

## Caveats of family-based filtering

While rare, it is possible that the assumed genetic model, under which the linkage or IBD analysis has been performed, may not be correct. This would lead to an incorrect filter. This can occur and is of particular concern for consanguineous pedigrees where it is usual to infer an autozygous recessive model. However, the inbreeding may be a coincidence, with the true mutation being most likely an incompletely penetrant dominant mutation or two compound heterozygous recessive mutations. The deeper the inbreeding loop, the less likely it is that an affected individual will still share a segment of an ancestor that is homozygous by descent, as indicated by an increasing LOD score for such regions in affected individuals, as parents are more distantly related. Extensive inbreeding may also make mapping results difficult to interpret, especially in the presence of locus heterogeneity (Markus et al. 2012).

The trio design for HTS projects is an extremely attractive approach for detecting hypothesized de novo variants in a cohort of sporadic cases where DNA from both parents is available. Without additional evidence for inbreeding in the population of origin, which suggests the possibilities of an autozygously inherited variant, the de novo genetic model is the most likely genetic model for singleton affected individuals and provides a powerful filter for variants. The trio design for de novo analysis is another family-based study design but it does not make use of linkage information, instead using the family data to eliminate inherited variants. Again, however, the success of this approach relies heavily on the hypothesized inheritance model being correct. In Taft et al. (2013), the initially hypothesized de novo genetic model was proven incorrect, with the discovery of two causal variants constituting a compound recessive model. However, if a de novo model is true, a linkage-based strategy would remove the causal variant from consideration as it constitutes a Mendelian error. This is the danger of using sporadics or even small families: occasionally the proposed genetic model may be incorrect. Thus, we propose that if de novo discovery fails both an autozygous mapping strategy and a compound heterozygote filtering model are applied. In addition, a linkage analysis can be performed with unaffected siblings. One unaffected sibling in a linkage analysis with a recessive model will remove ¼ of the genome from consideration, in the absence of inbreeding.

## Conclusions

Here, we have shown how IBD sharing analysis can be used in tandem with HTS to ensure data veracity, to identify appropriate genetic models that facilitate genotype filtering and to make inferences about which individuals are likely to share the ancestral susceptibility haplotype according to the best-fitting genetic model at each locus.

HTS is increasingly being applied to high morbidity, rare, Mendelian disorders where only sporadic cases are observed due to individuals being unlikely, or unable, to reproduce. These types of cohorts usually lack familial information for linkage or IBD studies available, unless the affected individuals are the product of a consanguineous union, in which case a powerful linkage analysis is possible. Other studies can only avail themselves to small, underpowered pedigrees for linkage, however, there is no longer a requirement for a priori statistically significant linkage, since WES and WGS are genome-wide variant identification methods, with no additional costs involved to examine further regions in the genome. A filter based on using multiple regions identified by either linkage or IBD analysis can still be a powerful method for the reduction of the number of candidate variants.

Many of the approaches we have outlined will become even more powerful as WGS supersedes WES and other targeted sequencing approaches. WGS is known to have less bias overall than WES and thus may identify causal variants that have been missed with WES (Bamshad et al. 2011). WGS will enable the genotype calling for many of the HapMap SNPs that can be further supplemented with 1000 Genome SNPs and SNVs leading towards the ultimate SNP chip. This will also lead to an increased ability to uniformly sample SNPs derived from HTS data, further improving linkage and IBD approaches.

HTS is not fail-safe. True causal variants may fail to be detected due to insufficient coverage or erroneous data (Koboldt et al. 2010). Current practice now sees coverage assessments for known genes post-WES and targeted capture screens of known disease causing genes (Carvill et al. 2013). Different laboratories use different criteria by which to ascribe success in a gene hunting effort. Hence, it is very difficult to know the failure rate of current studies with varying reports of success and even more difficult to determine false positive rates. It is important to delineate between the discoveries of novel genes versus novel variants in known genes. The latter solves a family but does not constitute a new basic research finding of high impact, yet it is an important clinical diagnostic success. Researchers searching for novel genes for disorders in cohorts of patients that have already undergone unsuccessful targeted sequencing for known genes have a much lower chance of success in finding a causal variant but are more likely to make a high impact research finding once the genetic cause of disease is identified. Thus, IBD and linkage approaches are likely to play a more crucial role in novel gene discovery, however, even targeted approaches will benefit from QC methodology that allows inference on relatedness and the detection of sample swaps and contamination.

The forthcoming improvements in HTS will lead to increased read length. These will improve our capacity to infer CNVs, microsatellites, other structural variations and facilitate de novo assembly and in silico haplotyping, which we have described above to also aid variant filtering. Detection of these types of variants in HTS data will also benefit from the use of family-based information. PennCNV (Wang et al. 2007) utilizes trio and quartet pedigree information to improve CNV calls for Illumina and Affymetrix SNP chip data and is currently being extended to HTS. Similarly, FamSeq (Peng et al. 2013) and PolyMutt (Li et al. 2012) improve variant calling by making use of familial information.

With the decreasing costs of HTS larger cohorts of affected individuals will be sequenced and some of these individuals will be related, either cryptically or by a known pedigree. Despite the undoubted success of HTS studies, the incorporation of familial information has the potential to enhance our efforts in this fast-moving field.
